# How Hospitals Help the Englishman Abroad

**Published:** 1911-06-18

**Authors:** 


### How Hospitals Help the Englishman Abroad.

### HOSPITAL PROGRESS IN HONG-KONG.

From a Correspondent.

After an absence of three decades from my native land, to which I returned a few years ago, I was much struck by the general advance here of medical science, and more especially by the progress made by hospitals. Not only had existing institutions been brought up to date and supplied with all the latest comforts and improvements, but new and vast buildings had arisen, in which patients were attended with a care and nursed with a skill undreamed of in the days of my youth, and the great sum of human suffering found a mitigation unhoped for in the early part of the nineteenth century. Hospital Sunday, with its splendid contribution to the funds of the various hospitals, had been successfully founded, and has flourished ever since. Whilst all these great and beneficial changes were taking place in the dear homeland, the healing art was also undergoing gradual expansion in the distant dependencies of the Empire, which soon participated in the discoveries, the improvements, and the developments of Western science.

Nowhere, perhaps, has a greater transformation taken place in the hospital accommodation than in the little colony of Hong-Kong. When I first arrived there the only institutions existing were the Government Civil Hospital, where all civil cases among the European and Indian population were treated, a Naval Hospital at Bowrington, on a breezy little hill at the east end of the city of Victoria, and the Tung Wa Hospital, built by the Government for Chinese and managed by a Chinese committee. The Civil Hospital, established on a rather ill-chosen site adjoining the Central Police Station in Hollywood Road, was inadequate in size, ill supplied with doctors, and entirely dependent on Chinese "boys" for attendance. Those patients who were unfortunate enough to belong to the floating population, and could not be tended by private practitioners, had to make the best of the Civil Hospital, its poor nursing, indifferently prepared food, and the necessarily limited medical attendance the hard-worked Superintendent could afford time and energy to give them. The Naval Hospital, efficiently run on old-fashioned methods, was of course confined to the use of the Fleet. The Tung Wa Hospital, which was opened in 1872 (with a flourish of trumpets) by the then Governor, Sir Richard Graves Macdonnell, and handed over to the control of a nominated body of Chinese, who promptly utilised their position to establish a sort of *imperium in imperio*, to influence their countrymen, became a sort of inferior poorhouse, where indigence was relieved and cases of sickness treated by empirical remedies which too often reduced it to a mortuary. The wards were dingy and dirty to a degree, the attendants ignorant and callous, the doctors the veriest quacks.

But as years rolled on changes were at length effected, on the initiative of the Colonial Surgeon and his subordinates. The old Civil Hospital in Hollywood Road was burned down in the great fire of 1878, when 365 buildings were reduced to ashes, and the Lock Hospital in Seyingpoon was enlarged and adapted for the purposes of a general hospital, the staff was then increased, and the building was at length (during the administration of Sir George Bowen) greatly enlarged and improved in 1884-85, though still maintained on the old site. Finally, through the urgent representations of the medical profession, strongly backed by the Colonial Press, nursing sisters were imported. At first French Sisters of Mercy were engaged, but owing to their lack of knowledge of English these helpers were soon exchanged for British nurses, who have ever since been employed, with the greatest success and to the entire satisfaction of the patients. In one of the annually recurring outbreaks of plague, the winter of 1898, two of the Sisters belonging to the Civil Hospital, who had volunteered to work in the plague mat-sheds, fell victims to their heroic labours, contracting the disease from the Chinese. Their self-sacrifice is commemorated by a stained-glass window in St. John's Cathedral.

Meantime other hospitals had arisen. The Alice Memorial Hospital, in Hollywood Road, was erected by Dr. Ho Kai, barrister-at-law, now C.M.G. and senior member of the Legislative Council, in memory of his wife, an English lady he married in England. This institution, for the benefit of Chinese, has been conducted efficiently ever since on English lines, and the Hong-Kong College of Medicine, now affiliated to the Hong-Kong University, at present in course of erection, was first established in connection with this hospital and the Nethersole Hospital on the Bonham Road, another private institution built and endowed by a benevolent former resident for the reception of Chinese who were willing to submit to Western methods. The college has already turned out a number of young Chinese licentiates, some of whom have made quite a reputation in the adjoining Chinese provinces.

The gradual rise into favour of Western medicine and practice among the Chinese had in the latter end of the nineteenth century encouraged the Colonial medical officers to direct the attention of the Colonial Government to the condition of the Tung Wa Hospital and the need for reform in its administration and for inspection by the head of the Medical Department, with the result that the Committee were induced to agree to the appointment of a Chinese medical officer who had been trained in Western medicine as house-surgeon, and to a complete reform of the treatment in and condition of the wards. So satisfied were the Committee with the improvements effected that they shortly after undertook the erection of a large new wing to the hospital, which has been built under European supervision on the latest sanitary conditions, and the patients are now treated, according to desire, on either Western or native methods, but in both cases are well tended and fed in cheerful wards, where cleanliness is maintained and light and air freely provided. This new wing was opened in 1903, during the administration of Sir Henry Blake, whose portrait in oils adorns the reception hall. About the same period the temporary mat-shed hospital for Chinese plague patients having become unsuitable, a well-designed, permanent structure was erected in Kennedy Town, and this has, I regret to say, been more or less in use every year since that date, the pestilence having proved of annual recurrence. It is to be hoped, however, that this disease will eventually die out or that some effective cure may be discovered for it.

Great as these developments must be admitted to be in a colony like Hong-Kong, they do not exhaust the list. The Victoria Jubilee Hospital, built on Barker Road, on the hills at the back of the city, some 1,400 feet above it, was opened to the public on November 7, 1903. This hospital, more especially designed for the benefit of European women and children, was at first little availed of, but is now increasingly useful for maternity cases, etc.

Another institution, still more recently erected on Mount Kellett, on a point some fifteen hundred feet above the sea, and opened in 1906, is the Matilda Hospital, built and endowed under the will of the late Mr. Granville Sharp, a former resident of the Colony, where he spent half a lifetime. This is more in the nature of a sanatorium for European and American residents in Hong-Kong and the Treaty ports of China, where they can secure gratuitous treatment and rest during convalescence. Mr. Sharp devoted the whole of his fortune—more than a million dollars—to the provision of this institution for the needy among the white communities, and named it after his wife, who pre-deceased him. It occupies a breezy site open to the south-west breezes, and has restored many a jaded invalid from the muggy and fever-haunted Treaty Ports of Southern China to health and strength.

I ought not to omit mention of the fact that a comfortable though small private hospital, known as the Peak Hospital, has been erected at Victoria Gap by the medical practitioners of the Colony for the use of paying patients. This is usually fully occupied by residents from the Treaty Ports.

The large increase in the garrison of Hong-Kong rendered necessary by the extension of its territory in 1898, when a territory of some 376 square miles was added to the Colony, naturally led to the provision of increased hospital accommodation. Previous to that time the principal hospital was housed in a roomy hulk moored near the centre of the harbour. This has now been put out of commission, and a large and handsome pile has been erected on a spacious site above the Bowen Road, some 300 to 400 feet above the various European barracks in Victoria. Medical provision for the Indian contingents who are quartered in Kowloon is made in that dependency.

From the foregoing brief sketch it will be seen that medical activity in the Ultima Thule of British power in the Far East, no less than in these islands, its centre and place of inception, reigns and flourishes. It will be gratifying to the philanthropic and the friends of medical science throughout the United Kingdom to know that the zeal of the savant and the scientist keeps pace with the ardour of the explorer in every part of the wide Empire.

